# A study of anaesthesia-related cardiac arrest from a Chinese tertiary hospital

**DOI:** 10.1186/s12871-018-0593-6

**Published:** 2018-09-11

**Authors:** Chu-Lian Gong, Jing-Ping Hu, Zhuo-Lin Qiu, Qian-Qian Zhu, Zi-Qing Hei, Shao-Li Zhou, Xiang Li

**Affiliations:** Department of Anesthesiology, The Third Affiliated Hospital of Sun Yat-sen University, Guangzhou, People’s Republic of China

**Keywords:** Anaesthesia, Cardiac arrest, Incidence

## Abstract

**Background:**

The present survey evaluated the incidence of perioperative cardiac arrests in a Chinese tertiary general teaching hospital over ten years.

**Methods:**

The incidence of cardiac arrest that occurred within 24 h of anaesthesia administration was retrospectively identified in the Third Affiliated Hospital of Sun Yat-Sen University between August 2007 and October 2017. Overall, 152,513 anaesthetics were included in the study period. Data collected included patient characteristics, American Society of Anaesthesiologists (ASA) physical status score, surgical specialty and anaesthesia technique. Cardiac arrests were assigned to one of three groups: “anaesthesia-related”, “anaesthesia-contributing” or “anaesthesia-unrelated”.

**Results:**

In total, 104 cardiac arrests (6.8:10,000) and 34 deaths (2.2:10,000) were obtained. Among them, eleven cardiac arrests events were anaesthesia-related, resulting in an incidence of 0.7 per 10,000 anaesthetics. Sixteen cardiac arrests events were found to be anaesthesia-contributing, resulting in an incidence of 1.0 per 10,000 anaesthetics. Cardiovascular adverse events were the major events that contributed to anaesthesia-related cardiac arrest. Differences were found between events related and unrelated to anaesthesia with regard to ASA physical status and anaesthesia technique (*P* < 0.05).

**Conclusions:**

Anaesthesia-related cardiac arrest occurred in 11 of 104 cardiac arrests within 24 h of anaesthesia administration. Most cardiac arrests related to anaesthesia were due to cardiovascular events, including arrhythmia and hypotension after intravenous narcotic, as well as haemorrhage. ASA physical status of at least 3 and subarachnoid block appeared to be relevant risk factors for anaesthesia-related cardiac arrest.

## Background

Since 2012, more than 310 million major surgical procedures have been performed annually worldwide [1]. Perioperative cardiac arrest is a rare but potentially catastrophic event that is associated with high mortality. An overall perioperative cardiac arrest rate of 7.19/10,000 anaesthetics was reported worldwide from the 1990s to the 2000s [2]. In recent decades, several studies have described the incidence and causes of postoperative cardiac arrest related to anaesthesia in different patient populations from different countries, such as the United States [3], Germany [4], France [5], Pakistan [6], Saudi Arabia [7], and Brazil [8].

Although more than 10% of major surgical procedures worldwide are performed in China [1], there is limited information available on the incidence of anaesthesia-related cardiac arrest and risk factors for perioperative cardiac arrest in Chinese tertiary hospitals. Most Chinese publications regarding anaesthesia-related cardiac arrest are published in Chinese journals that are not indexed by the global databases. A multicentre prospective survey, including 106,569 patients from eleven Chinese teaching hospitals, found the incidence of cardiac arrest for patients undergoing regional anaesthesia to be 0.09/10,000 [9]. However, studies on perioperative cardiac arrest for large unselected patient populations are still lacking. As the incidence of cardiac attest within the 24 h perioperative period is an important component of the anaesthesia quality control index system published by the National Health and Family Planning Commission of the People’s Republic of China in 2015, we sought to assess the incidence and risk factors for anaesthesia-related cardiac arrest within the 24 h perioperative period in a Chinese tertiary care university hospital.

## Methods

This project was approved by the Research Ethics Committee of the Third Affiliated Hospital of Sun Yat-sen University (Ref: [2017] 2–216). Because of the retrospective and anonymous nature of this study, written informed consent was waived by the Research Ethics Committee of the Third Affiliated Hospital of Sun Yat-sen University. We retrospectively analysed data from critical incident reports of our Department of Anaesthesiology from 152,513 anaesthesiological procedures at the Third Affiliated Hospital of Sun Yat-sen University from August 2007 to October 2017.

The Third Affiliated Hospital of Sun Yat-sen University, which was founded in 1971, is an 1800-bed public tertiary teaching hospital performing more than 15,000 surgeries per year to all ages and provides care to the population of Guangdong province and the surrounding areas. In our department, it is mandatory to record critical incidents, including cardiac arrest, that occur within 24 h of anaesthesia administration in an anaesthesia database. This record is compiled and completed by the anaesthesia team involved in the anaesthetic case.

In accordance with previous studies [3, 4, 8], cardiac arrest was defined as an event requiring cardiopulmonary resuscitation, which might involve closed- or open-chest compressions.

According to the classification system from Hohn et al. [4], all cardiac arrest events were assigned to one of three groups based on the contributory factor that caused the cardiac arrest: anaesthesia-related group (anaesthesia was the only or major contributing factors); anaesthesia-contributing group (both surgery and anaesthesia were the contributing factors or there was some doubt whether cardiac arrest was entirely attributable to anaesthesia); and anaesthesia-unrelated group (surgery or other factors were the contributing factors) (Table 1).

**Table 1:** Classification system for cardiac arrest [4]

Group	Definition
Anaesthesia-related	Where it is reasonably certain that CA was caused by the anaesthesia or other factors under the control of the anaesthetist
Anaesthesia-contributing	1. Where there is some doubt whether CA was entirely attributable to the anaesthesia or other factors under the control of the anaesthetist 2. Where CA was caused by both surgical and anaesthesia factors
Unrelated to anaesthesia	1. CA where the administration of the anaesthesia did not contribute and surgical or other factors are implicated 2. Inevitable CA, which would have occurred irrespective of anaesthesia or surgical procedures 3. Incidental CA, which could not reasonably be expected to have been foreseen by those looking after the patient, was not related to the indication for surgery and was not due to factors under the control of the anaesthetist or surgeon. 4. Those that cannot be assessed despite considerable data but where the information is conflicting or key data are missing 5. Cases that cannot be assessed because of inadequate data

For each cardiac arrest case, basic characteristics of the patient (name, age, sex); surgical procedures (elective, urgent or emergency surgery) and area; American Society of Anaesthesiologists (ASA) physical status classification; anaesthetic technique (general anaesthesia, regional anaesthesia including epidural/spinal/caudal or plexus block, sedation); and a checklist of airway, respiratory, cardiocirculatory, neurological, renal and miscellaneous events were obtained.

To avoid a potential incomplete case collection, the anaesthesia team that was responsible for each cardiac arrest event was asked to review the case and provide a written summary and presentation for peer review. The cardiac arrest commission of the Department of Anaesthesiology at the Third Affiliated Hospital of Sun Yat-sen University, which was composed of three senior anaesthesiologists, analysed the anaesthesia and medical records, critical incident report form, written summary and presentation for each cardiac arrest event. Disagreements on the cause of cardiac arrest were resolved by discussion among the three members, and agreement or consensus was determined when at least two out of three members agreed on the event cause.

The primary adverse events leading to cardiac arrest that occurred within 24 h of anaesthesia administration were grouped into the following categories, as proposed by Cheney et al.: respiratory (difficult intubation, inadequate ventilation/oxygenation, oesophageal intubation, premature extubation, aspiration, airway obstruction, endobronchial intubation, bronchospasm, and inadvertent extubation), cardiovascular (multifactorial/miscellaneous events, pulmonary embolism, inadequate fluid therapy, stroke, haemorrhage and myocardial infarction), medication-related, equipment-related, block-related, procedural, iatrogenic and other not further classified incidents [10]. In addition, for the cardiovascular category, arrhythmia and hypotension were involved in multifactorial/miscellaneous events in circumstances where the primary event leading to cardiovascular system changes was not obvious. Also included in the multifactorial cardiovascular events were surgical complications and patient conditions, including tamponade, and pathologic abnormalities that were undiagnosed before surgery but determined by autopsy, such as congenital abnormalities, viral myocarditis, myocardial fibrosis, and unsuspected severe coronary artery disease [10].

The characteristics of anaesthesia-related or contributed cardiac arrest cases and anaesthesia unrelated cardiac arrest cases were summarized and compared. We used means and SDs for continuous variables and numbers and percentages for categorical variables. The χ^2^ test and two independent samples t-test were used to compare categorical and continuous variables, respectively. Statistical analysis for all data was performed using SPSS software (version 20.0, SPSS, Chicago, IL, USA). A *P* value of less than 0.05 was considered statistically significant.

## Results

**Fig. 1:**
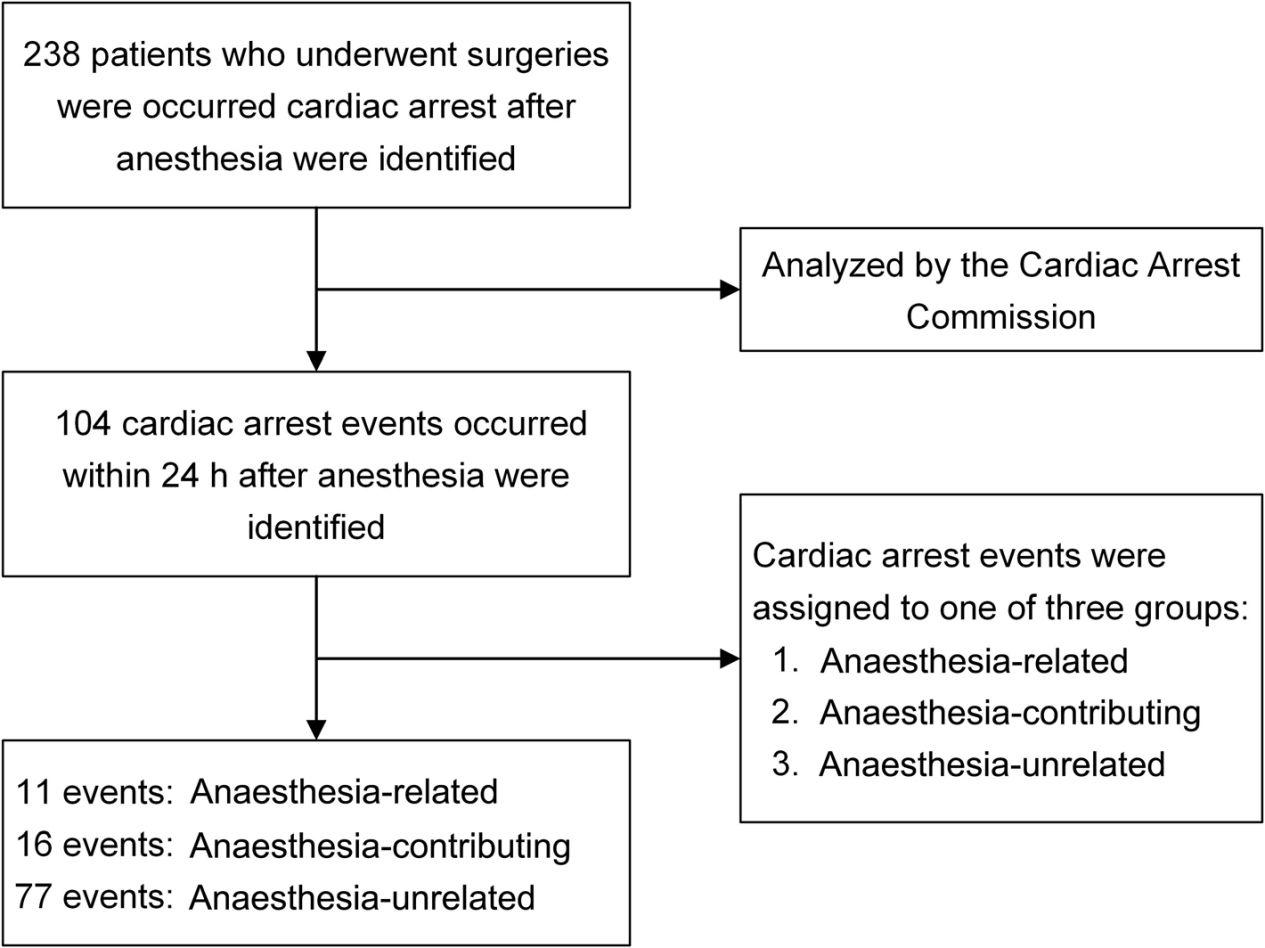
Flow diagram of review process to identify anaesthesia-related and anaesthesia-contributing cardiac arrest events

Over the 10 years of the study (2007–2017), 152,513 patients received anaesthesia care at the Third Affiliated Hospital of Sun Yat-sen University. Within this time period, 238 patients who underwent surgery experienced cardiac arrest after anaesthesia administration. Among those patients, 104 cardiac arrest events occurred within 24 h of anaesthesia administration, which meant that the cardiac arrest rate within 24 h of anaesthesia administration was 6.8/10,000. The overall mortality from cardiac arrest within 24 h of anaesthesia administration was 44 of 104 cardiac arrest events (2.9/10,000). Among 11 patients with cardiac arrest related to anaesthesia, four (36.4%) did not survive, while for cardiac arrests that contributed to or were unrelated to anaesthesia, six (6/16, 37.5%) and 34 (34/77, 44.2%) patients died, respectively. Figure 1 shows a flow diagram illustrating the review process for identifying cardiac arrest events.

For all 104 patients with cardiac arrest, the median age was 52 years old (range 2 to 96 years old). Only four patients with cardiac arrest were less than 3 years old, while 15 patients were older than 75 years. Males comprised 57.7% of the cardiac arrest cases (60/104). Forty patients with cardiac arrest were ASA physical status of at least 3, and 25 cardiac arrest patients had a New York Heart Association (NYHA) functional score of at least 3. Fifty-three cardiac arrest patients underwent emergency surgeries. General anaesthesia was the predominant anaesthetic technique used in cases with cardiac arrest.

Patient characteristics and adverse events leading to anaesthesia-related cardiac arrest are shown in Table 2. There were 11 cardiac arrest events, resulting in a cardiac arrest rate related to anaesthesia of 0.7 per 10,000 anaesthetics. The median age was 50 years old (range 2 to 96 years old). Males comprised 54.5% of the anaesthesia-related cardiac arrest cases. General anaesthesia was the primary technique. Four patients with anaesthesia-related cardiac arrest died (36.4%), which meant a mortality rate of 0.3/10,000. Cardiovascular adverse events were the major events contributing to cardiac arrest (*n* = 4), which comprised 36.4% of the cases. Among these four cases, three of the patients were older than 85 years. In addition, medication, respiratory and regional block-related adverse events comprised the remaining cases.

**Table 2:** Patient characteristics and adverse events leading to anaesthesia-related cardiac arrest (*n* = 11)

No.	Age range	Sex	NYHA	Speciality	ASA PS	Anesthesia Technique	Adverse Event Leading to Cardiac Arrest	Category	Outcome
1	90–100	1	III	Orthopedic surgery	IV	GA	Hypotension and dysrhythmia after intravenous narcotic. Multiple comorbidities.	Cardiovascular	Recovered
2	50–60	2	I	General surgery	IIIE	GA	Dysrhythmia due to the use of neophryn for hypotension and bradycardia during surgery.	Medication	Recovered
3	90–100	2	III	Spinal surgery	III	GA	Bradycardia and dysrhythmia after intravenous narcotic. Multiple comorbidities.	Cardiovascular	Died
4	30–40	2	I	Traumatology	IIIE	GA	Massive aspiration of blood after induction, hypoxia.	Respiratory	Died
5	20–30	2	I	Spinal surgery	I	SAB	Bradycardia and dysrhythmia after postural change.	Regional block	Recovered
No.	Age range	Sex	NYHA	Speciality	ASA PS	Anesthesia Technique	Adverse Event Leading to Cardiac Arrest	Category	Outcome
6	20–30	1	I	ENT surgery	I	GA	Loss of airway on PACU due to bleeding and laryngospasm after nasal trumpet placed.	Respiratory	Recovered
7	40–50	2	II	Traumatology	IIIE	GA	Intraperitoneal hemorrhage, cardiac arrest during insertion of central venous catheter.	Cardiovascular	Died
8	30–40	1	I	General surgery	I	SAB	Bradycardia and dysrhythmia 15 min after the block.	Regional block	Recovered
9	80–90	1	III	Orthopedic surgery	IV	GA	Hypotension and acute myocardial ischemia after intravenous narcotic. Multiple comorbidities.	Cardiovascular	Died
10	70–80	1	II	Orthopedic surgery	III	SAB	Seizure and dysrhythmia due to the local anesthetic intoxation.	Medication	Recovered
11	0–10	1	I	ENT surgery	I	GA	Displacement of endotracheal tubes during surgery. A failure to ventilate and intubate. Problem with fixation of the tracheal tube.	Respiratory	Recovered

*NYHA* New York Heart Association, *ASA PS* American Society of Anesthesiologists physical status score, *GA* general anesthesia, *SAB* subarachnoid block, *PACU* postanesthesia care unit

Table 3 shows the patient characteristics and adverse events leading to anaesthesia-contributing cardiac arrest. The number of anaesthesia-contributing cardiac arrests was 16, which is a rate of 1.0/10,000. Six patients (37.5%) with anaesthesia-contributing cardiac arrest died, which meant a mortality rate of 0.4/10,000. The median age was 55 years old (range 3 to 86 years old). Males comprised 56.3% of the anaesthesia-related cardiac arrest cases. The majority of patients (75.0%, 12/16) suffered from cardiac arrest due to cardiovascular complications, which included myocardial infarction, hypotension, ST segment depression, bradycardia and ventricular fibrillation. There were three cases (18.8%) of respiratory complications, all of which had a cardiac arrest after arrival in the intensive care unit (ICU) or post-anaesthesia care unit (PACU).

**Table 3:** Patient characteristics and adverse events leading to anaesthesia-contributory cardiac arrest (*n* = 16)

No.	Age range	Sex	NYHA	Speciality	ASA PS	Anesthesia Technique	Adverse Event Leading to Cardiac Arrest	Category	Outcome
1	50–60	2	I	General surgery	II	GA	Intraoperative hemorrhage and hyperkalemia with inadequate volume resuscitation during the case.	Cardiovascular	Recovered
2	60–70	1	II	Thoracic surgery	III	GA	Hypotension and bradycardia 15 min after induction of anesthesia. Biopsy showed pericardial tamponade induced by pericardial metastatic tumor	Cardiovascular	Died
3	40–50	1	II	General surgery	II	GA	Haemorrhagic shock due to lesion of the artery. Recurrent episodes of hypotension. Problems with intraoperative management.	Cardiovascular	Recovered
4	50–60	2	I	Neurosurgery	IIIE	GA	Unstable angina and severe ST segment depression before surgery. Cardiac arrest 10 min after induction of anesthesia.	Cardiovascular	Died
No.	Age range	Sex	NYHA	Speciality	ASA PS	Anesthesia Technique	Adverse Event Leading to Cardiac Arrest	Category	Outcome
5	70–80	1	III	Traumatology	IIIE	GA	Respiratory arrest 30 min after arrival in ICU. Likely cause respiratory arrest secondary to multiple rib fractures, pulmonary contusion and paradoxical respiratory movement.	Respiratory	Recovered
6	50–60	2	II	Vascular surgery	IVE	GA	Aorta abdominalis embolism and severe hyperkalemia (potassium value ≥7.5 mmol/L) before surgery. Persistent hypotension and arrhythmia after induction of anesthesia. Ventricular fibrillation 25 min after induction of anesthesia.	Cardiovascular	Died
7	40–50	1	I	Gynecologic surgery	II	GA	Bradycardia and hypotension after the administration of pituitrin.	Medication	Recovered
No.	Age range	Sex	NYHA	Speciality	ASA PS	Anesthesia Technique	Adverse Event Leading to Cardiac Arrest	Category	Outcome
8	50–60	1	II	General surgery	II	GA	Laparoscopic hepatectomy. Hypotension and arrhythmia after 50 min of surgery incision. Likely cause cardiac arrest secondary to intraoperative pulmonary embolism.	Cardiovascular	Died
9	50–60	2	II	General surgery	II	GA	Intraoperative hemorrhage and ventricular fibrillation. Problems with intraoperative management.	Cardiovascular	Recovered
10	50–60	2	II	General surgery	II	GA	Intraoperative hemorrhage and hypotension. Inadequate volume replacement after intraoperative massive hemorrhage.	Cardiovascular	Recovered
11	80–90	2	III	Orthopedic surgery	III	GA	Respiratory arrest after extubation in PACU. Likely cause respiratory arrest secondary to the blocking of respiratory tract by sputum.	Respiratory	died
No.	Age range	Sex	NYHA	Speciality	ASA PS	Anesthesia Technique	Adverse Event Leading to Cardiac Arrest	Category	Outcome
12	50–60	2	II	Traumatology	IIIE	GA	Intraoperative hemorrhage and ventricular fibrillation. Problems with intraoperative management.	Cardiovascular	Recovered
13	20–30	1	I	Gynecologic surgery	II	GA	Respiratory arrest 10 min after arrival in PACU. Postoperative respiratory depression secondary to narcotics administered throughout case and within 30 min of extubation in the OR.	Respiratory	Recovered
14	60–70	2	II	General surgery	II	GA	Intraoperative hemorrhage and hypotension. Inadequate volume replacement after intraoperative massive hemorrhage.	Cardiovascular	Recovered
15	80–90	2	III	General surgery	IIIE	GA	Bowel obstruction and recent history of MI. Probably inadequate volume resuscitation.	Cardiovascular	Died
16	0–10	1	II	Cardiac surgery	III	GA	Pulmonary vasospasm and hypertension 1 h after arrival in ICU. Likely due to severe vomiting and aspiration.	Cardiovascular	Died

*NYHA* New York Heart Association, *ASA PS* American Society of Anesthesiologists physical status score, *GA* general anesthesia, *SAB* subarachnoid block, *ICU* intensive care unit, *PACU* postanesthesia care unit

As the four paediatric patients occupied a small number of the 104 patients undergoing cardiac arrest within 24 h of anaesthesia administration, the univariate analyses for risk factors were only performed on adult patients. Differences were found between events related and unrelated to anaesthesia with regard to ASA physical status and anaesthesia technique (Table 4, all *P* < 0.05), while no differences were found between events contributing to and unrelated to anaesthesia (Table 5).

**Table 4:** Univariate analysis for risk factors of adult patients with anaesthesia-related cardiac arrest

Terms	Anaesthesia related to cardiac arrest		Anaesthesia unrelated to cardiac arrest		*P*
	n	Mean ± SD or percentage	n	Mean ± SD or percentage	
Age (yr)	10	56.5 ± 28.3	75	53.2 ± 17.4	0.725
Mortality	4	40.0%	34	45.3%	0.750
Sex					0.936
Male	6	60.0%	44	58.7%	
Female	4	40.0%	31	41.3%	
ASA PS					0.019
< 3	3	30.0%	51	68.0%	
≥ 3	7	70.0%	24	32.0%	
NYHA					0.752
< 3	7	70.0%	56	74.7%	
≥ 3	3	30.0%	19	25.3%	
Anaesthesia technique					0.008
GA	7	70.0%	71	94.7%	
SAB	3	30.0%	4	5.3%	
Surgical characteristics					0.492
Emergency	3	30.0%	44	58.7%	
Non-Emergency	7	70.0%	31	41.3%	

*ASA PS* American Society of Anaesthesiologists physical status score, *NYHA* New York Heart Association, *GA* general anaesthesia, *SAB* subarachnoid block

**Table 5:** Univariate Analysis for risk factors of adult patients with anaesthesia-contributing cardiac arrest

Terms	Anaesthesia contributing to cardiac arrest		Anaesthesia unrelated to cardiac arrest		*P*
	n	Mean ± SD or percentage	n	Mean ± SD or percentage	
Age (yr)	15	58.3 ± 15.5	75	53.2 ± 17.4	0.290
Mortality	6	40.0%	34	45.3%	0.704
Sex					0.924
Male	9	60.0%	44	58.7%	
Female	6	40.0%	31	41.3%	
ASA PS					0.275
< 3	8	53.3%	51	68.0%	
≥ 3	7	46.7%	24	32.0%	
NYHA					0.661
< 3	12	80.0%	56	74.7%	
≥ 3	3	20.0%	19	25.3%	
Anaesthesia technique					0.360
GA	15	100.0%	71	94.7%	
SAB	0	0.0%	4	5.3%	
Surgical characteristics					0.072
Emergency	5	33.3%	44	58.7%	
Non-Emergency	10	66.7%	31	41.3%	

*ASA PS* American Society of Anesthesiologists physical status score, *NYHA* New York Heart Association, *GA* general anaesthesia, *SAB* subarachnoid block

## Discussion

During the perioperative period, cardiac arrest and death always represent the worst patient outcomes and are still the most severe challenges for anaesthetists. From the 1990s to the 2000s, the global incidences of perioperative cardiac arrest ranged from 6.59/10,000 anaesthetics in highly developed countries to 20.68/10000 in less-developed countries [2]. In the last decades, China has experienced significant improvements in economic and human indicators, thereby decreasing the inequality in relation to countries with very high human development. The present study reported comparable incidences of overall and anaesthesia-related cardiac arrests in a Chinese tertiary hospital over a ten-year period (2007–2017) to high human development countries (according to the Human Development Index (HDI) set by the United Nations Development Programme), such as the United States [3], Germany [4] and Brazil [11].

The adverse events leading to anaesthesia-related cardiac arrest differ among various studies. Although the respiratory and airway-related adverse events are considered the major reasons for anaesthesia-related fatal outcomes (death, cardiac arrest) [3, 4], this has not been consistent for all studies. The rates of anaesthesia-related death resulting from airway management events have ranged widely from 7.9 to 80% [12]. A study based on data from the Pediatric Perioperative Cardiac Arrest Registry revealed that cardiovascular events, including hypovolaemia from blood loss and hyperkalaemia from transfusion of stored blood, were the most common causes for anaesthesia-related cardiac arrest (41% of all cardiac arrest patients) [13]. In the present study, cardiovascular events and problems were also the primary cause of anaesthesia-related cardiac arrest (36.4% of all anaesthesia-related cardiac arrest patients), and respiratory, medication and regional block accounted for the rest of the events. Some analyses have demonstrated that the predominance of cardiovascular events in anaesthesia-related cardiac arrest may be associated with the increasing use of respiratory monitors, such as pulse oximetry, capnography, disconnection alarms, and low-pressure alarms, which may be more helpful to prevent respiratory rather than cardiovascular events [10, 11]. In addition, advances in clinical practices, such as adoption of standardized guidelines for management of difficult airways, might also be helpful for reducing the incidence of cardiac events due to the airway [14].

Most patients experiencing anaesthesia-related cardiac arrest due to cardiovascular events and problems were older than 85 years, and the cardiac arrest occurred after intravenous narcotic. This result was in accordance with the study reported by Nunes et al., who found that two-thirds of anaesthesia-related cardiac arrest events in older patients were also due to cardiovascular collapse after neuroaxial anaesthesia [15]. In this study, three elderly patients had multiple serious cardiovascular comorbidities, including hypertension, coronary heart disease and arrhythmia; thus, they were particularly vulnerable to cardiovascular events, such as persistent hypotension and myocardial infarction due to the neuroaxial anaesthesia. Previous studies have suggested an adequate preoperative evaluation that might be helpful for avoiding the incidence of anaesthesia-related cardiac arrest [16–18]. Therefore, adopting perioperative medical practices with demonstrable effectiveness, organizing multidisciplinary discussion of adverse effects and implementing evidence-based safety protocols are necessary for preventing anaesthesia-related cardiac arrests in older patients.

Notably, two anaesthesia-related cardiac arrest events were due to regional block problems. Cardiac arrest events during spinal anaesthesia are rare and unexpected but are not uncommon. The incidence of cardiac arrest after spinal anaesthesia and neuraxial blockade was reported to range from 1.3 to 18 per 10,000 anaesthetic [17]. In this study, both of patients were young and healthy, and ropivacaine was used for subarachnoid injection to obtain maximum sensory block up to the T6 level. Two patients developed bradycardia (heart rate < 30/min) and subsequently were unresponsive with asystole 15–20 min after spinal anaesthesia without any prodromal symptoms. However, the mechanism that triggers cardiac arrest under spinal anaesthesia remains controversial and unclear. The contribution of intrinsic cardiac mechanisms and autonomic imbalance with the background of parasympathetic predominance might provide a more convincing and physiologic explanation for the occurrence of abrupt severe bradycardia and cardiac arrest under spinal anaesthesia [19, 20]. Furthermore, over sedation, respiratory arrest, unintentional total spinal, myocardial infarction and local anaesthetic toxicity might also attribute to the causative factors [18]. Fully understanding the physiologic changes caused by spinal anaesthesia and its complications, appropriately selecting patients, respecting the contraindications of the procedure, performing adequate monitoring, and exhibiting constant vigilance are particularly important for the eventual outcome [19, 20].

In this study, 15 adult patients were in the anaesthesia-contributing group. Most of them were due to cardiovascular events, which was consistent with the reports from the Germany tertiary care university hospital by Hohn et al. [4]. It had been suggested that the mortality for adult anaesthesia-contributing cardiac events was much higher than that for anaesthesia-related cardiac events [3]. Our study showed that the mortality for anaesthesia-contributing cardiac events was 40.0% (6/15), which was comparable to that of anaesthesia-related cardiac events. This result might be related to the more complicated intraoperative events in these cases that may be caused by anaesthesia, surgery, or other factors.

The limitations of our study are as follows. First, the retrospective nature of the present study is a great limitation; hence, a prospective study will help to clarify the findings. Second, risk factors were only identified from the population of patients undergoing cardiac arrest within 24 h of surgery. Thus, the present analysis based on patients who were likely at risk of cardiac arrest within the 24-h perioperative period may not necessarily be generalized to the entire population of 152,513 patients. Third, the results of the present study were from a single-centre study, which might not be generalizable. We hope to perform a multi-centre prospective survey to reveal the incidence and risk factors for anaesthesia-related cardiac arrest.

## Conclusions

In summary, we found eleven anaesthesia-related cardiac arrest cases of 104 cardiac arrests within 24 h of anaesthesia administration. Most cardiac arrests related to anaesthesia were due to cardiovascular events, including arrhythmia and hypotension after intravenous narcotic, as well as haemorrhage. In addition, ASA physical status of at least 3 and subarachnoid block appeared to be relevant risk factors for anaesthesia-related cardiac arrest. We hope the results of this study will serve as a basis for national benchmarking.

## Abbreviation

ASA: American Society of Anesthesiologists
